# Determinants of Renal Tissue Oxygenation as Measured with BOLD-MRI in Chronic Kidney Disease and Hypertension in Humans

**DOI:** 10.1371/journal.pone.0095895

**Published:** 2014-04-23

**Authors:** Menno Pruijm, Lucie Hofmann, Maciej Piskunowicz, Marie-Eve Muller, Carole Zweiacker, Isabelle Bassi, Bruno Vogt, Matthias Stuber, Michel Burnier

**Affiliations:** 1 Department of Nephrology, University Hospital, Lausanne, Switzerland; 2 Department of Nephrology and Hypertension, Bern University Hospital, Bern, Switzerland; 3 Department of Radiology, Medical University of Gdansk, Gdansk, Poland; 4 Department of Radiology, University Hospital, Lausanne, Switzerland; University Medical Center Utrecht, Netherlands

## Abstract

Experimentally renal tissue hypoxia appears to play an important role in the pathogenesis of chronic kidney disease (CKD) and arterial hypertension (AHT). In this study we measured renal tissue oxygenation and its determinants in humans using blood oxygenation level-dependent magnetic resonance imaging (BOLD-MRI) under standardized hydration conditions. Four coronal slices were selected, and a multi gradient echo sequence was used to acquire T2* weighted images. The mean cortical and medullary R2* values ( = 1/T2*) were calculated before and after administration of IV furosemide, a low R2* indicating a high tissue oxygenation. We studied 195 subjects (95 CKD, 58 treated AHT, and 42 healthy controls). Mean cortical R2 and medullary R2* were not significantly different between the groups at baseline. In stimulated conditions (furosemide injection), the decrease in R2* was significantly blunted in patients with CKD and AHT. In multivariate linear regression analyses, neither cortical nor medullary R2* were associated with eGFR or blood pressure, but cortical R2* correlated positively with male gender, blood glucose and uric acid levels. In conclusion, our data show that kidney oxygenation is tightly regulated in CKD and hypertensive patients at rest. However, the metabolic response to acute changes in sodium transport is altered in CKD and in AHT, despite preserved renal function in the latter group. This suggests the presence of early renal metabolic alterations in hypertension. The correlations between cortical R2* values, male gender, glycemia and uric acid levels suggest that these factors interfere with the regulation of renal tissue oxygenation.

## Introduction

Numerous experimental studies have suggested that disturbed oxygenation plays a role in the development and progression of kidney disease including hypertensive nephropathy[1]–[3].Thus, using micro-electrodes for direct pO2 measurements, low cortical O2 levels have been found in spontaneous hypertensive rats and in rats with streptozotocine-induced diabetes or in the subtotal nephrectomy model [4], [5].

Until recently, human data were largely lacking mainly due to the lack of non-invasive methods to estimate renal tissue oxygenation. Over the last decade, blood oxygenation level- dependent magnetic resonance imaging (BOLD-MRI) has become a powerful tool to estimate renal tissue oxygenation non-invasively in humans. The basic principle of BOLD-MRI is that changes in renal tissue desoxyhemoglobin concentrations involve generation of phase incoherence of magnetic spins, leading to an increase in apparent relaxation rate R2* (expressed in sec^−1^). Under the assumption that blood pO2 is in equilibrium with tissue pO2, R2* values provide estimates of tissue oxygenation, a low R2* indicating a high tissue oxygenation [6]. Using post-processing programs, several circles - called regions of interest (ROI's) - are placed manually per slice in the cortex and in the medulla. This allows the assessment of the average cortical and medullary R2* value, per kidney or for both kidneys together, without the need to administer contrast product [7].

Several studies have used BOLD-MRI in humans to investigate renal oxygenation in different forms of chronic kidney disease (CKD) [8], [9]. The largest study which assessed renal oxygenation at different degrees of kidney dysfunction was published by Michaely et al and included 400 patients [10]. Interestingly, no correlation was found between cortical and medullary R2* values and the estimated glomerular filtration rate (eGFR, according to the MDRD-formula) in this study. However this evaluation had several limitations: renal function was not measured in all patients (280 with measured creatinine levels), no information was collected on medication intake or baseline characteristics such as blood pressure or underlying renal disease, and BOLD-MRI was not performed under standardized conditions of fluid and sodium intake which are known to have a strong influence on renal oxygenation measured by BOLD-MRI [11], [12].

Changes in renal tissue oxygenation may also contribute to the development of ischemic and hypertensive nephropathies [5]. For these reasons, most studies performed in hypertensive patients so far have been conducted in patients with renal artery stenosis [13], [14]. Other studies in patients with essential hypertension have focused on the role of sodium, angiotensin II or race on cortical and medullary oxygenation [12], [15], [16]. These studies were performed in a relatively small number of participants, or did not include a control group. Whether or not essential hypertension is characterized by chronic hypoxia in humans remains therefore an open question.

The aim of the present study was therefore to assess renal tissue oxygenation at baseline and after an acute administration of furosemide in patients with various levels of renal function as well as in patients with essential hypertension and a normal renal function and in control subjects. Moreover, we analyzed the potential determinants of renal cortical and medullary tissue oxygenation in these patient groups.

## Methods

This research project was approved by the local institutional review committee (Ethical Committee of the Canton de Vaud, Switzerland) and conducted according to the principles expressed in the Declaration of Helsinki. Written informed consent was obtained from each participant.

### Subjects

Patients with CKD stage 1–5, or with hypertension without CKD were eligible for this study. CKD was defined as an estimated glomerular filtration rate (eGFR) ≤60 ml/min/1.73 m^2^, or the presence of structural or functional abnormalities for at least three months [17]. Arterial hypertension (AHT) was defined as mean office blood pressure (BP) ≥140/90 mmHg measured at more than one occasion, or an office BP <140/90 mmHg while taking one or more antihypertensive drugs. Other inclusion criteria were: age ≥18 years and the ability to understand the study protocol and to sign an informed consent. Controls were normotensive, untreated healthy individuals without a history of kidney disease or hypertension or any other concomitant disease. Exclusion criteria for all participants were: a contra-indication to MR-imaging such as claustrophobia or the presence of a pacemaker or other implanted metallic device.

### Study protocol

Patients were recruited at the outpatient nephrology and hypertension clinic of the university hospital in Lausanne (CHUV). Controls were recruited by local advertisement. Participants were maintained on their regular diet. Salt intake, proteinuria, and creatinine clearance were measured before BOLD-MRI by a 24 h urine collection. On the day of each BOLD-MRI measurement, an identical oral hydration protocol was followed by each participant at home (loading dose of 5 ml/kg of water at 8am, followed by 3 ml/kg every hour till 12am), see extended methods for further details and justification of this hydration protocol. Subjects joined our research unit at 11.30 am. BP was measured three times by an experienced research nurse using an automated Omron 705IT oscillometric device according to the recommendations of the European Society of Hypertension [18]. Peripherical oxygen saturation was measured using a fingertip pulse oxymeter (Oxy, Medair AB, Delsbo Sweden), and blood was drawn by a catheter inserted into an antecubital vein.

### BOLD-MRI

**Figure 1:**
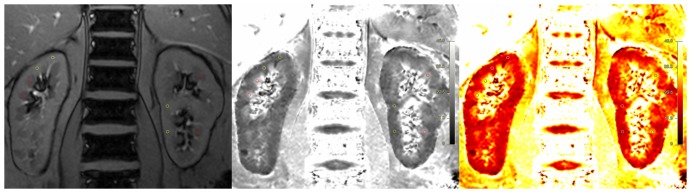
Illustration of BOLD image analysis. Anatomical templates are shown on the left, R2* maps in the middle, and color maps on the right (low R2* levels corresponding to higher tissue oxygenation in red, high R2* levels in yellow). Two regions of interest (ROIs) are traced in the form of circles (20 voxels each) in the cortex and medulla of each kidney; this procedure is repeated on four different slices.

BOLD-MRI was performed between 1 and 2 pm in the radiology department. Magnetic resonance measurements were carried out on a 3T whole-body MR system (MAGNETOM Trio, Siemens Medical Systems, Erlangen, Germany), as described previously [12], [19], [20]. Briefly, four coronal slices with good cortico-medullary differentiation obtained in expiration were selected from morphological images for functional evaluation with BOLD-MRI. Twelve T_2_*-weighted images were recorded within a single breath-hold of 12.4 seconds with a modified Multi Echo Data Image Combination sequence (MEDIC) with the following parameters: repetition time (TR) 68 ms, echo time (TE) 6–52.2 ms (equidistant echo time spacing 4.2 ms), flip angle 20°, field of view (FOV) 400×400 mm^2^, voxel size 1.6×1.6×5 mm^3^, bandwidth 700 Hz/pixel, matrix 256×256 (interpolation to 512×512). All images were exported for analysis with a home-built IDL program (Interactive Data Language, Boulder, CO, USA). R_2_* maps were calculated voxel by voxel using a Levenberg-Marquardt least-squares algorithm to fit an exponential function to the signal intensities measured for each echo time. ROIs were traced in the form of circles of equal size (containing approximately 20 voxels each) in the medulla and the cortex (two in the cortex and two in the medulla in each kidney), as illustrated in Figure 1. The reported R_2_* value was the mean value of four slices (16 ROIs) for the medulla and the cortex. This technique has been shown to have a good reproducibility [7], [20]. Medullary-cortical R_2_*ratio (MCR2*), as a marker of metabolic workload, was calculated for each participant by dividing the mean medullary R_2_* by cortical R_2_* levels [21]. The procedure was repeated for all four coronal series obtained fifteen minutes after the administration of 20 mg furosemide intravenously.

### Statistics

Clinical data were analyzed using STATA 11.0 (StataCorp, College Station, Texas, USA). Quantitative variables were expressed as mean ± standard deviation, or as median (25^th^–75^th^ percentile range), as appropriate. Qualitative variables were expressed as number of patients and percentage. Comparisons between baseline characteristics of study groups were analyzed with ANOVA. Distribution of variables was also expressed using the probability density function according to Kernel. In case of non-normal distribution, variables were log-transformed. Multivariable logistic regression, adjusting for the predefined variables age, sex, current smoking, body mass index, diabetes, hemoglobin, and 24 h urinary sodium excretion, was used to determine the independent association of cortical and medullary R2* values with CKD status. These variables were selected based on their known association with CKD (body mass index, diabetes, age), or because of a theoretical association with renal tissue oxygenation (hemoglobin, 24 h urinary sodium excretion, smoking).

Similarly, in order to determine the independent association of each independent variable of interest with the dependent variables cortical and medullary R2* values, multivariate linear regression was performed, adjusted for the same predefined covariates.

Associations between the eGFR slope and R2* values were examined with Spearman's rank correlation and multivariable linear regression analysis. Results of the logistic multivariate analysis are presented as Odds-ratio (OR) and 95% confidence interval (95% CI). Results of all linear multivariate analyses are presented as beta-coefficients (β) and their 95% confidence intervals. *P* values were derived from maximum likelihood ratio tests. Statistical significance was considered for a two-sided *P*<0.05.

## Results

### Clinical characteristics of the patients and controls

Details of the screening procedure are provided in the 'extended Methods and Results S1' section. Baseline clinical characteristics of patients with CKD and hypertension and of controls are shown in Table 1 . Patients in the CKD-and hypertensive group were well-matched for age, sex and other baseline characteristics. Healthy volunteers were significantly younger, and more often female. Medical treatments of subjects according to group are shown in Table 2 and across different stages of CKD in Table S1.

**Table 1:** Baseline characteristics of patients and controls enrolled in the study.

	Control (n = 42)	CKD (n = 95)	AHT (n = 58)
Age (years)	46±13*	56±15	57±11
Sex (% female)	52*	30	32
Currently smoking (%)	7*	27	35
Body Mass Index (kg/m2)	26±5	27±5	29±5
Systolic BP (mmHg)	122±13*	135±19	142±16**
Diastolic BP (mmHg)	73±10	76±12	82±10**
eGFR (CKD-EPI, ml/min/1.73 m^2^)	97±14*	57±31	91±15**
eGFR (MDRD, ml/min/1.73 m^2^)	94±15*	57±29	91±16**
Hemoglobin (g/dl)	136±11	130±18	138±13**
Blood glucose (mmol/l)	5.6±0.9*	6.5±2.1	6.1±1.2
Diabetes (%)	0*	23	17
Blood potassium (mmol/l)	3.9±0.2*	4.2±0.6	3.8±0.3**
Venous bicarbonate (mmol/l)	27(20;30)*	25 (14;32)	27 (23;32)**
Blood uric acid (μmol/l)	289 (130;450)*	391 (168;662)	342 (163;548)**
Oxygen saturation (%)	97±2.0	96±1.9	96±1.6
24 h Urinary volume (ml)	1731 (694;5008)	2068 (585;4356)	1812 (780;3945)
24 h Urinary sodium excretion (mmol)	156±72	173±92	174±87
24 h Urinary protein excretion (g)	0.06 (0;0.12)	0.3 (0;9.4)	0.07 (0;0.17)
24 h Urinary albumin excretion (mg)	4.7 (0;23)	100 (1;6131)	10 (0;29)
24 h Urinary creatinine clearance (ml/min)	125 (83;213)	68 (14; 170)	115 (73;249)

Values are expressed as mean±SD, or as median (min; max) as appropriate. CKD = chronic kidney disease; AHT =  arterial hypertension.

* p<0.05: control group versus CKD;

** p<0.05 AHT versus CKD.

**Table 2:** Drug treatment of participants.

	CKD (n = 95)	AHT (n = 58)	Controls (n = 42)
*Antihypertensive medication (%)*			
Beta blocker	34.9	39.6	0
Alpha blocker	2.3	0	0
ACE-inhibitor	17.4	11.5	0
AT-II type 1 receptor blocker	52.3	26.4	0
Calcium channel blockers	32.6	35.9	0
Thiazide diuretic	27.9	20.8	0
Loop diuretic	17.4	3.9	0
*Cholesterol lowering medication*			
Statine	57	33	5
Fibrate	1.2	0	0
Ezetimibe	5.8	9.6	0
*Antiplatelet agent*			
Aspirin	31.4	41.5	2.4
*Uric acid lowering medication*			
Allopurinol	17.4	3.9	0
*Vitamin D*	23.3	3.9	0
*Antidiabetic medication*			
Oral antidiabetics	8.1	11.5	0
Insuline	17.4	3.8	0

All values are expressed as the percentage of patients of each group treated with the drug in question; CKD: chronic kidney disease (CKD), AHT: arterial hypertension AHT.

### R2* levels in hypertension and CKD as compared with healthy controls

**Figure 2:**
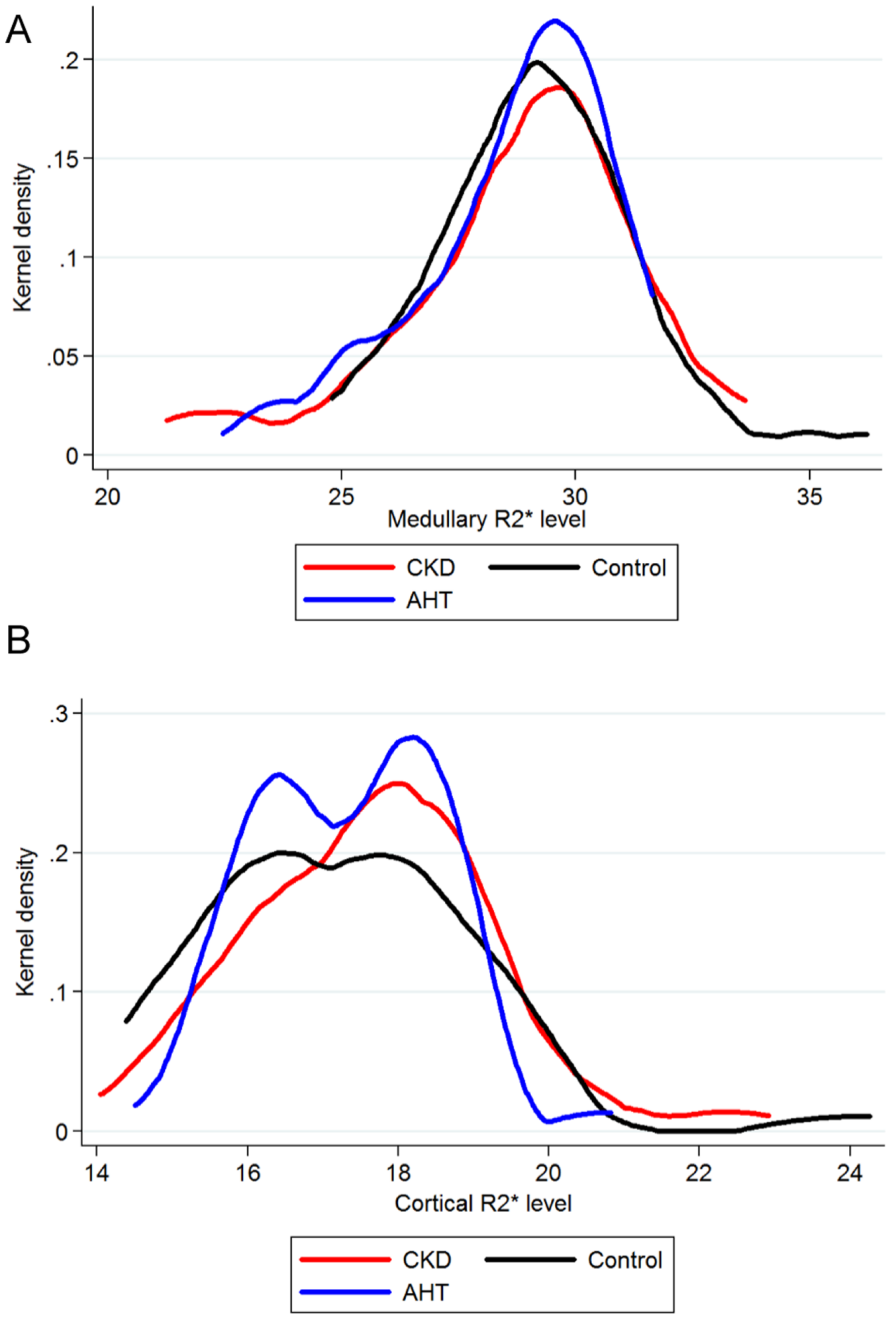
Probability density function (Kernel density) of renal R2* values. A/medullary R2* values and B/cortical R2* values.

**Figure 3:**
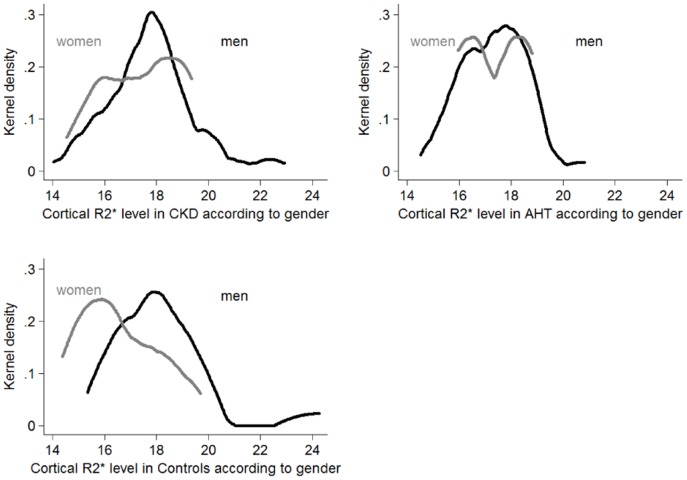
Probability density function (Kernel density) of cortical R2* values by gender, according to group.

**Figure 4:**
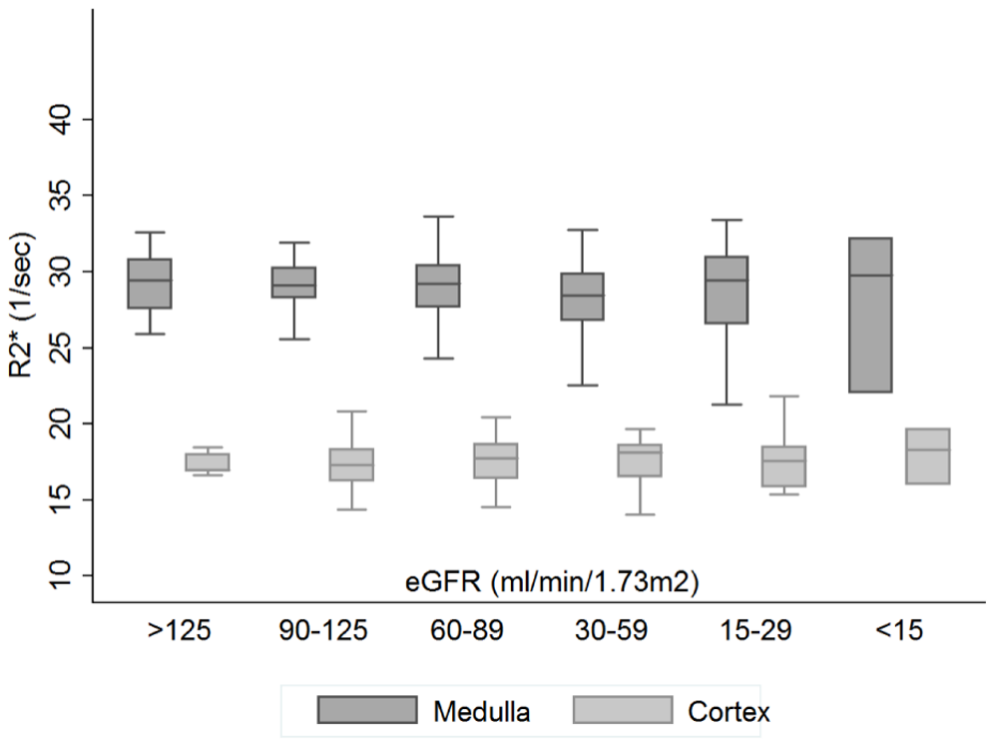
Medullary and cortical R2* values over decreasing eGFR_mdrd_ values. The number of subjects was n = 10 (for the eGFR>125 ml/min/1.73 m^2^ category), 64 (eGFR 90–125), 65 (60–89), 36 (30–59), 15 (15–29) and 5 (<15), respectively.

There were no differences in mean or median cortical and medullary R2* levels between the right and left kidney in all groups (cortex (median (range)) respectively 17.4 (16.1; 18.6) and 17.6 (16.3; 18.8) sec^−1^, p = 0.29; and medulla (mean±SD): 28.7±4.2 vs 28.7±2.5 sec^−1^, p = 0.94). Therefore, only the mean cortical and medullary R2* values of both kidneys are shown and used for statistical analysis. The distribution of medullary R2* was comparable in all groups, but that of cortical R2* values differed markedly between groups (Figure 2A and B). A bimodal cortical distribution was observed in controls and AHT patients, yet not in CKD patients (Figure 2B). The bimodality of the probability density function of cortical R2* values was partly explained by gender (Figure 3); no gender-differences were seen in the distribution of medullary R2* values (Figure S1). The mean medullary and cortical R2* values and medullary-cortical ratio's of each group were comparable between the CKD, hypertensive and control group (Table 3). No differences were seen in cortical and medullary R2* levels between stage I–V of CKD (Figure 4 and Figure S2 for results by gender).

**Table 3:** Baseline medullary and cortical R2*values and medullary cortical ratio's (MCR), overall and by gender.

	Control	CKD	AHT	p (ANOVA)
Baseline Medullary R2*	**29.3±2.4**	**28.8±2.6**	**28.6±2.1**	**0.44**
men	29.5±2.7	29.1±2.6	28.7±2.1	0.53
women	29.0±1.7	28.3±2.7	28.4±2.2	0.50
Furosemide-induced Change medullary R2*	**−6.1±2.9**	**−3.7±2.4**	**−4.7±2.1**	**<0.001**
men	−6.8±3.3	−3.7±2.5	−4.9±2.1	0.001
women	−5.4±2.2	−3.7±2.1	−4.2±2.1	0.02
Baseline Cortical R2*	**17.1 (15.9;18.7)**	**17.6 (16.5;18.6)**	**17.4 (16.3;18.3)**	**0.27**
men	17.8 (16.9;19.0)	17.8 (17.0;18.5)	17.4 (16.4;18.2)	0.36
women	16.2 (15.3;17.8)*	17.5 (16.1;18.7)	17.4 (16.0;18.6)	0.18
Furosemide-induced Change cortical R2*	**−1.2 (−0.83;−1.52)**	**−1.2 (−0.58;−1.8)**	**−1.3 (−0.86;−1.92)**	**0.36**
men	−1.0 (−0.88;−2.2)	−1.2 (−0.55;−1.8)	−1.3 (−1.0;−1.78)	0.81
women	−1.2 (−0.52;−1.5)	−1.5 (−0.59;−2.0)	−1.9 (−0.9;−2.4)	0.08
Baseline MCR ratio R2*	**1.70±0.2**	**1.62±0.2**	**1.65±0.1**	**0.11**
men	1.64±0.2	1.62±0.2	1.66±0.1	0.71
women	1.76±0.1	1.63±0.16	1.64±0.13	0.01
MCR ratio R2* after furosemide	**1.44±0.2**	**1.51±0.2**	**1.50±0.1**	**0.21**
men	1.35±0.2	1.51±0.2	1.48±0.11	0.06
women	1.54±0.2*	1.52±0.1	1.55±0.16	0.98

All values are shown before and after the administration of intravenous furosemide. Subjects on loop diuretics (n = 2 in the AHT group and n = 15 in the CKD group) are not included in the furosemide-induced changes.

Values expressed in sec^−1^, as mean±SD or as median (25^th^–75^th^ percentile), as appropriate. CKD = chronic kidney disease; AHT =  arterial hypertension. * P<0.05 concerning within-group differences between men and women.

**Figure 5:**
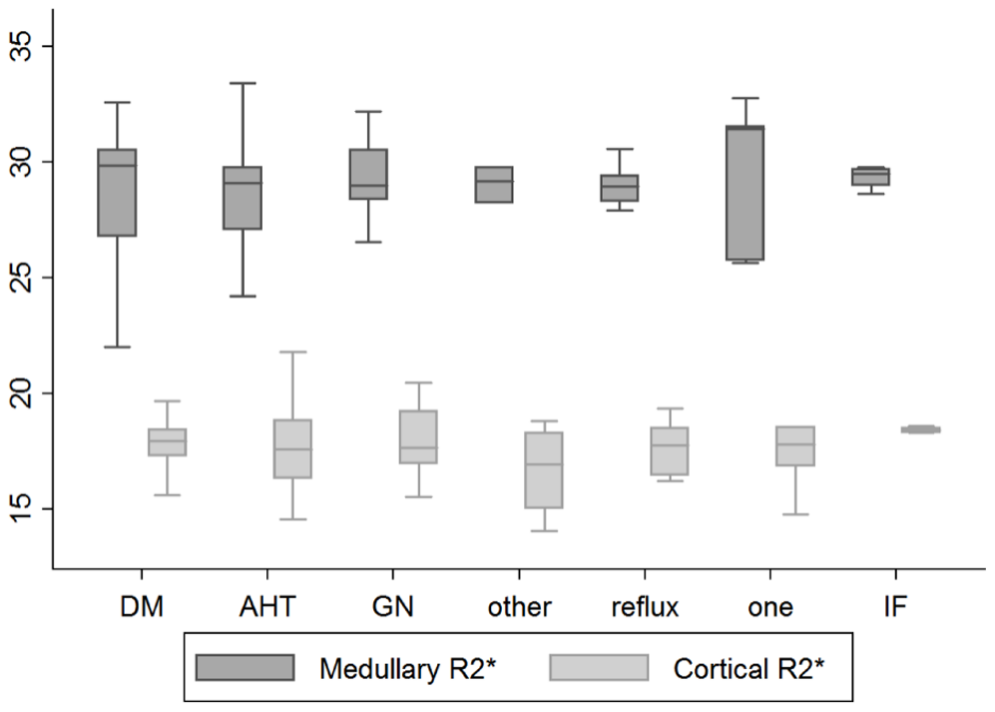
Cortical and medullary R2* values according to the cause of CKD. Values shown as boxplots; No (no CKD, n = 100), DM (diabetic nephropathy, n = 20), AHT (hypertensive nephropathy, n = 31), GN(glomerulonephritis, n = 17), reflux (reflux nephropathy, n = 6), one (solitary kidney, n = 7), IF (interstitial nephritis, n = 6), other (other cause of kidney disease, n = 8). There were no differences between cortical and medullary R2* levels between the groups (ANOVA p = 0.10 and p = 0.99, respectively)

In addition, within the CKD group no significant difference was observed according to the renal diagnosis (Figure 5).

### Changes in R2* after furosemide in hypertension or CKD patients as compared with controls

**Figure 6:**
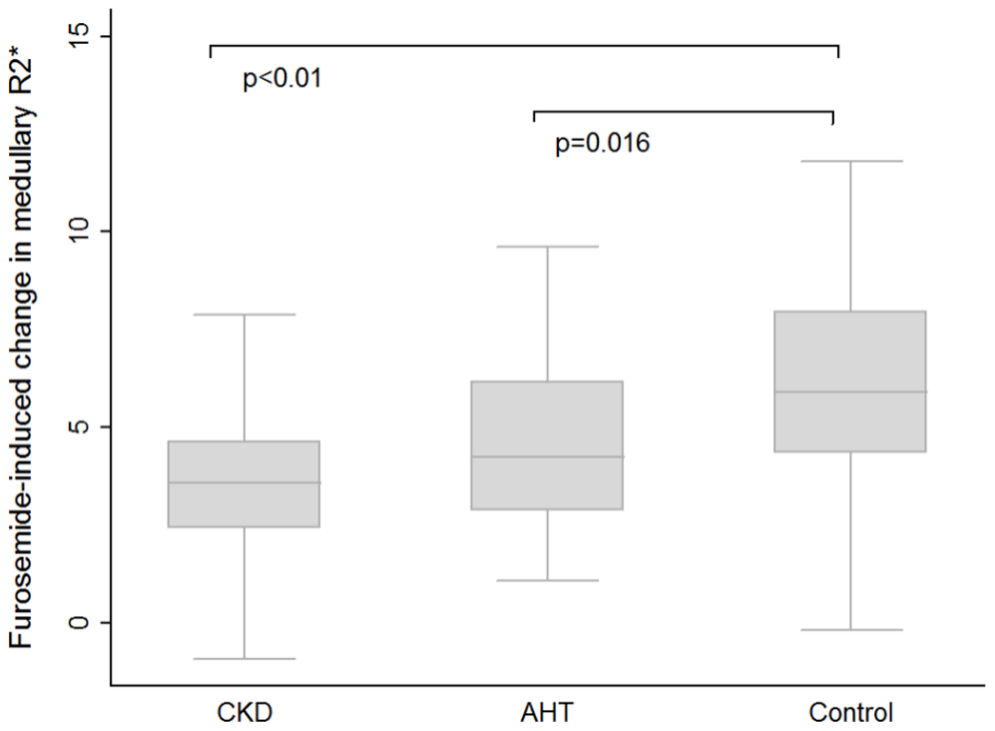
Furosemide-induced change in medullary R2* values according to group.

The situation was different in acute conditions, since the response to furosemide differed significantly between groups. Medullary R2* but not cortical values decreased significantly (suggesting an increase in medulla oxygenation) in the three groups, both in men and women (Table 3) but the reduction was significantly smaller in the CKD and AHT groups (Figure 6). Although there was a trend towards larger furosemide-induced decreases in medullary R2* in men, this difference was not statistically significant. In age-and gender adjusted multivariable linear regression analysis the furosemide-induced change in medullary R2* (f-R2*) correlated significantly with eGFR_mdrd_ (adjusted β per ml/min/1.73 m^2^: −0.03 (95% CI 0.01; 0.05), p = 0.003); the latter association persisted when intake of loop diuretics was introduced in the model (see extended Methods and Results S1).

The difference in furosemide-induced change between the AHT and control group was not explained by a difference in eGFR. In multivariate logistic regression adjusted for age, sex, and eGFR_mdrd,_ the furosemide-induced change in medullary change in R2* level remained significantly smaller in the presence of AHT (OR 0.81; 0.64–0.98, p = 0.047).

### Associations between renal tissue R2* levels, CKD status, hypertension, and kidney function

In multivariate logistic regression, adjusted for age, sex, body mass index, hemoglobin, current smoking and urinary sodium excretion, with CKD status as dichotomized dependent variable (CKD compared with healthy controls and hypertensive subjects pooled together), medullary R2* levels were not associated with CKD status (OR(95% CI): 1.01 (0.86;1.18), p = 0.92). Medullary R2* levels were also not associated with CKD status when comparing the CKD group separately with healthy controls (CKD vs controls, OR: 0.95 (0.79; 1.15), p = 0.59), or with the hypertensive group (CKD vs. AHT, OR: 1.1 (0.91;1.35), p = 0.3).

Although cortical R2* levels were higher in CKD patients, this difference was not statistically significant in adjusted models (CKD versus non-CKD, OR: 1.20 (0.95; 1.51, p = 0.14)). The same trend was seen when comparing the CKD group separately with healthy controls (OR: 1.10 (0.85; 1.41), p = 0.49).

The determinants of renal cortical and medullary oxygenation were also analyzed in the entire population using linear models. There was no association between medullary R2* levels and eGFR or any of the other predefined variables in the adjusted multivariable linear regression analysis (Table 4A). No significant difference was observed when antihypertensive treatment (renin-angiotensin system (RAAS) blockers or antihypertensive treatment in general) was added to the model. Similar results were found for the association between medullary R2* values and creatinine clearance as measured by 24 h urinary collection (adjusted β per ml/min: −0.01, p = 0.96).

**Table 4:** Multivariate linear regression analysis examining correlations between baseline characteristics and medullary and cortical R2* levels.

	Medullary R2*		Cortical R2*	
**A/**	**β^1^**	***P***	**β^1^**	***p***
Sex (female vs. male)	−0.43	0.49	−1.8	*0.01*
Age (per year)	−0.01	0.72	−0.01	0.7
BMI (per kg/m2)	−0.03	0.61	−0.04	0.58
eGFR (MDRD)	−0.003	0.75	−0.01	0.29
Smoking (yes vs. no)	0.28	0.77	1.04	0.16
Urinary 24 h sodium excretion (mmol)	0.002	0.52	0.004	0.13
Diabetes (yes vs. no)	−1.01	0.41	1.67	0.07
**B/**	**β^1^**	***P***	**β^1^**	***p***
Mean Arterial BP (per mmHg)	−0.04	0.036	0.003	0.91
Urinary 24 h protein excretion (per g)	−0.18	0.58	−0.39	0.32
RAAS-blocker (yes vs.no)	−0.86	0.11	0.34	0.6
Serum glycemia (per mmol/l)	−0.11	0.54	0.91	<0.001
Serum uric acid (per μmol/l)	−0.0001	0.76	0.014	<0.001

Correlations are expressed as regression coefficient β. Correlations between R2* levels and predefined factors are shown under (A). The analysis including the additional variables glycemia, serum uric acid level and 24 h urinary proteinuria in the model is shown under (B).

1 adjusted for gender, age, BMI, eGFR, smoking, urinary sodium excretion, Hemoglobin, and diabetes.

Results for cortical R2* levels and its associations with predefined variables were also largely negative (Table 4A). There was a positive association between gender and R2* levels (adjusted β −1.8, p = 0.01). Hence, male participants had higher cortical R2* levels, corresponding to lower cortical oxygenation.

Since only a small proportion of the variability in R2* levels was explained by the predefined variables, some additional analyses were performed on the entire population. Thus, a negative correlation was found between medullary R2* levels and mean arterial blood pressure (Table 4B). However, the correlation between mean arterial pressure (MAP) and medullary R2* values was weak and disappeared when including antihypertensive treatment in the model (β −0.04 per mmHg, p = 0.043 before, and β −0.03, p = 0.13 after adjustment for antihypertensive treatment). Results of multivariable linear regression analysis are shown in more detail in Table S2, S3, S4. Diabetic subjects (n = 32) had higher cortical R2* values than non-diabetic subjects (mean R2* 18.7±5.9 vs 17.5±1.6 sec^−1^, p = 0.05), yet diabetes was not associated with cortical oxygenation in adjusted logistic regression models (Table S3). In contrast, cortical R2* levels correlated positively and significantly with circulating glucose levels as measured just before BOLD-MRI (Table 4B and Table S4). Including antidiabetic treatments in the multivariate regression model did not alter this result.

The positive correlation found between uric acid and cortical R2* levels did not change after the inclusion of allopurinol and diuretic treatments, or mean arterial blood pressure in the model. Results for cortical R2* levels stratified by sex are shown in Table 5. Similar positive associations were found between cortical R2* levels and uric acid; the association between cortical R2* levels and glycemia were only present in men, whereas there was a positive association between cortical R2* and age in women yet not in men.

**Table 5:** Multivariate linear regression analysis examining correlations between baseline characteristics and cortical R2* levels, stratified by gender.

Cortical R2*	Men		Women	
	β°	*p*	β°	*p*
Age (per year)	−0.05	0.17	0.049	0.02
BMI (per kg/m2)	−0.07	0.57	−0.03	0.54
eGFR (MDRD)	−0.03	0.11	−0.002	0.81
Smoking (yes vs. no)	1.69	0.1	−1.06	0.16
Urinary 24 h sodium excretion (mmol)	−0.007	0.11	0.001	0.65
Diabetes (yes vs. no)	2.47	0.06	−0.63	0.46
Glycemia (per mmol/l)	1.13	<0.001	0.31	0.15
Uric acid (per mmol/l)	0.015	0.004	0.01	0.001

°adjusted for age, BMI, eGFR, smoking, urinary sodium excretion, Hb, and diabetes.

In order to assess whether some of the results were linked to an over-representation of young women in the control group, sensitivity analyses were performed in a subgroup excluding healthy women <40 years (n = 13) and men aged >75 years suffering from CKD (n = 5). The main results of the study remained unchanged (see Methods and Results S1).

## Discussion

The main findings of this study are that: 1) mean cortical and medullary R2* values as a proxy for renal tissue oxygenation are similar in hypertensive patients, CKD patients and healthy controls; however, the distribution of cortical R2* values differs markedly between groups, 2) the medullary R2* response to furosemide is blunted in hypertensive patients and markedly reduced in CKD patients, 3) baseline renal tissue oxygenation appears to be remarkably stable over different degrees of kidney dysfunction, independently of the cause of kidney disease and 4) cortical R2* levels are positively associated with male gender, glycemia and uric acid levels.

The first interesting observation of this paper is that although mean cortical and medullary R2* look identical in hypertensive and CKD patients and controls, the distribution of cortical R2* differs between groups with a clear bimodal distribution in controls and AHT and a unimodal shape of the distribution in CKD. As found in our population, part of the bimodal distribution is linked to the male/female ratio. However, this ratio is similar in hypertensives and CKD patients suggesting other mechanisms explaining the differences in distribution, which should be the subject of further study.

The second finding of our study is that renal cortical and medullary oxygenation as measured by BOLD-MRI appears to be extremely well maintained in patients with CKD. Indeed, in contrast to what has been observed experimentally we did not find any decrease in cortical or medullary oxygenation even in advanced CKD; the nature of the underlying renal disease does not appear to play a major role. In this respect, our data are in accordance with the study by Michaely et al [10], although the latter study could be criticized for some methodological issues. Despite the fact that our study has been performed under very standardized hydration conditions, and detailed information was available on possible confounders such as medication, sodium intake and the type of underlying kidney disease we were not able to demonstrate a reduction of tissue oxygenation as renal disease progressed. In addition, this study also found no alterations in renal R2* values in persons with essential hypertension in comparison with healthy controls, despite the fact that numerous previous animal studies have reported renal tissue hypoxia in AHT [5].

The discrepancy between animal studies and BOLD-studies in humans regarding oxygenation can be interpreted in different ways. First of all, it might be that BOLD-MRI is not sensitive enough or simply not as good a tool to assess renal tissue oxygenation in CKD-patients as is direct invasive measurements using microelectrodes. Nonetheless, early animal studies performed to validate the BOLD-MRI technique have reported linear relationships between directly measured renal pO2 values and the BOLD signal [22]. Alternatively, the ROI-technique used to analyze BOLD-images might be less applicable in CKD patients, where loss of cortico-medullary differentiation hampers manual placement of ROIs. This might play a role in the interpretation of medullary R2* values, but cortical R2* values are expected to be reliable, thanks to its anatomic proximity with the kidney capsula. Besides, the majority of subjects in the present study had less advanced stage I–III kidney disease, and largely maintained cortico-medullary differentiation; in patients with preserved differentiation, BOLD-MRI is a reliable, reproducible method. Nevertheless, it remains an open question if studies using different methods of analysis such as the recently described compartmental model [23] would obtain similar results. Assuming that BOLD-MRI is able to correctly estimate tissue oxygenation in humans, our findings together with those of Michaely et al put into question whether ‘chronic renal hypoxia’ truly exists in humans. In conditions of acute hypoxia, hypoxia-inducible factors (HIF) are stabilized, which stimulates erythropoietin production, angiogenesis and metabolic reprogramming, offering protection and increasing survival of renal tubular cells under hypoxic conditions [2], [24]. In contrast, chronic HIF stabilization stimulates interstitial fibrosis, a process that is generally believed to compress peritubular capillaries, which further decreases local tissue oxygenation and leads to a vicious circle of HIF-stabilization, increased formation of interstitial fibrosis, glomerulosclerosis and renal function decline [3], [25]. However, hypoxia-induced fibrosis may not be a vicious circle that further worsens oxygenation, but merely a way to maintain renal oxygenation by adapting oxygen consumption to the demand. Identical mechanisms might operate in animals. Animal models reporting hypoxia in CKD such as the 5/6 nephrectomy model may not have been carried out long enough to simulate the long-term adaptive changes in the kidney that might occur after several decades of chronic kidney disease or hypertension in humans. In line with this hypothesis, Priardarshy et al [26] evaluated renal oxygenation six to eight weeks after remnant kidney creation (instead of the 2 weeks applied by Manotham et al.)[1], and found that renal oxygenation was not decreased, but rather increased in the remnant kidney. A similar observation was made in renal artery stenosis with a reduction of renal tissue oxygenation acutely but a good maintenance of tissue oxygenation in the chronically stenotic kidney [14].

A role of renal handling of sodium in mediating oxygen consumption is supported by our observation that the medullary R2* response to furosemide differs between controls and hypertensives with a blunted response in hypertension and an even more marked reduction in CKD patients. In CKD, the markedly reduced response to furosemide can be explained by the reduced renal function leading to lower concentrations of furosemide within the kidney. However, this cannot be the explanation for hypertensive patients who had a comparable renal function as controls. Pratt et al have previously demonstrated ethnic differences in the response to furosemide [27]; to the best of our knowledge, differences in response to furosemide between normo-and hypertensive white subjects have not been reported previously. The blunted response to furosemide observed in hypertensive patients may be an indirect marker of the alterations in renal sodium handling in hypertension. Persons with essential hypertension have an increased proximal tubular reabsorption and a reduced distal delivery of sodium, which might blunt the effect of furosemide [28]. Alternatively, there might be differences in mitochondrial metabolism and oxygen consumption in the thick ascending limb of Henle between hypertensive and normotensive subjects, in analogy with recently described differences between Dahl salt-sensitive rats and salt-resistant control strains [29].

Our multivariate analysis enabled us to identify several new factors associated with renal tissue oxygenation. Thus, cortical R2* levels was positively and strongly associated with male gender. The relationship with male gender was robust and persisted in sensitivity analyses, and suggests that cortical oxygenation might be regulated differently in men and women. It may also provide some clues why renal function declines faster in men. However, our data do not offer any explanation for the higher R2* values in men, and studies measuring simultaneously renal perfusion and tubular sodium handling are necessary to clarify this issue.

Renal tissue hypoxia has been a consistent finding in mouse and rat models of diabetic nephropathy [4], [30]. The reason why this occurs is less certain, but oxydative stress and glomerular hyperfiltration leading to increased tubular sodium load and oxygen consumption have been proposed as the main mechanisms [31], [32]. BOLD-MRI studies in humans have shown mixed results, some studies reporting higher, others lower R2* values in patients with diabetes [8], [9], [33]. In this study, DM2 patients had slightly higher cortical R2* levels than controls, suggesting lower tissue oxygenation, yet this association was not maintained in adjusted models. Interestingly, cortical oxygenation correlated linearly and positively with glucose levels as measured just before the MRI exam, suggesting that glycemia directly or indirectly (via sodium transport) influences cortical oxygenation. In a study assessing the influence of renin-angiotensin blockers on renal oxygenation in patients with type 2 DM, we have recently reported similar results [19]. These data suggest that episodes of hyperglycemia might induce transient increases in oxygen consumption due to hyperfiltration which increases sodium transport in the proximal tubule [34]. The pre-MRI glycemia has not been systematically reported in previous studies, which might partly explain previous mixed results. The herein described association offers an alternative explanation in humans for to the relationship between badly controlled diabetes mellitus and adverse renal outcome [35]. However, the described relationship is based on a cross-sectional analysis which limits causal inferences, and needs confirmation in interventional studies.

Our observation that circulating uric acid levels correlate positively with cortical R2* levels adds information to the ongoing debate about the role of uric acid in cardiovascular disease. Hyperuricemia-induced cortical vasoconstriction could be partly responsible for the herein described correlation between uric acid and cortical hypoxia [36], but more studies are necessary to clarify this.

This study has some limitations. Firstly, no information was acquired on renal perfusion. We therefore cannot determine whether changes in oxygenation were the result of alterations in oxygen delivery. Nevertheless, Textor and colleagues have shown previously that renal tissue oxygenation is largely independent of renal blood flow, and that cortical (but not medullary) oxygenation only falls in case of severe renal artery stenosis occluding more than 90% of the lumen [13]. Another limitation of our study is its cross-sectional design. Finally, all measurements were performed without stopping concomitant medication, which might have weakened eventual underlying differences in R2* levels between the groups

## Conclusion

Our data suggest that renal tissue oxygenation at rest is comparable in controls, treated hypertensives and CKD patients. However, the response to furosemide differs between groups providing some insights on the mechanisms linking renal tubular function, oxygen consumption and renal function in hypertension and/or chronic kidney diseases. Our data do not support the concept that chronic kidney disease is associated with decreased renal tissue oxygenation in humans, as repeated observed in animal models. However, acute changes in oxygenation most likely occur in humans, as illustrated by the changes in R2* in response to furosemide. Future interventional studies should clarify the role of renal sodium handling, blood glucose and serum uric acid in the regulation of renal tissue oxygenation as newly described in this study. The differences in cortical R2* levels between men and women suggest that renal oxygenation is possibly regulated differently in men and women; this point also needs further study. Although no correlation was found between R2* values and (previous) kidney function, it is still possible that renal tissue R2* values predict kidney function decline but this is to be demonstrated in prospective studies.

## Supporting Information

Figure S1 Probability density function (Kernel density) of medullary R2* values by gender, according to group.
(TIF)

Figure S2 Medullary and cortical R2* values over decreasing eGFR_mdrd_ values, by gender. The number of subjects was n = 44 (for the eGFR>90 ml/min/1.73 m^2^ category), 41 (eGFR 60–90), 25 (eGFR 30–60), 15 (eGFR <30) in men and respectively 30,24,11, and 5 in women.
(TIF)

Table S1 **Drug treatment of patients across different stages of chronic kidney disease (CKD).**
(DOCX)

Table S2 Multivariate linear regression analysis examining associations between baseline characteristics and medullary R2* levels. Associations between medullary R2* levels and baseline characteristics are expressed as regression coefficient β (95% CI).
(DOCX)

Table S3 Multivariate linear regression analysis examining associations between baseline characteristics and cortical R2* levels. Associations between cortical R2* levels and baseline characteristics are expressed as regression coefficient β (95% CI).
(DOCX)

Table S4 Multivariate linear regression analysis examining correlations between baseline characteristics and medullary and cortical R2* levels. This analysis includes the additional variables glycemia, serum uric acid level and 24 h urinary proteinuria.
(DOCX)

Methods and Results S1 **Extended methods and results.** - Screening. -Methods: Justification hydration protocol. -Results: * Furosemide-induced change in R2* in patients already on diuretics. * Sensitivity analysis in subgroup excluding healthy women <40 years and male CKD patients >75 years.
(DOCX)
