# Supplementary figures and images for: Determinants of Renal Tissue Oxygenation as Measured with BOLD-MRI in Chronic Kidney Disease and Hypertension in Humans
Source: PLoS One. 2014 Apr 23;9(4):e95895. doi: 10.1371/journal.pone.0095895 (PMC3997480; doi:10.1371/journal.pone.0095895)

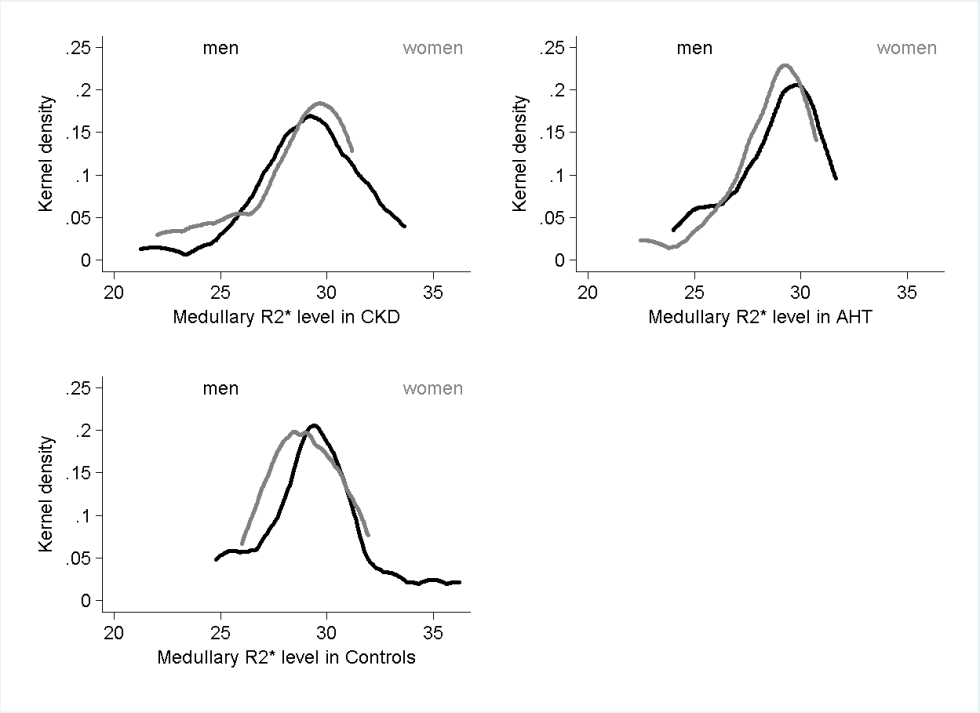

Supplement: Figure S1 — Probability density function (Kernel density) of medullary R2* values by gender, according to group. (TIF) [file pone.0095895.s001.tif]

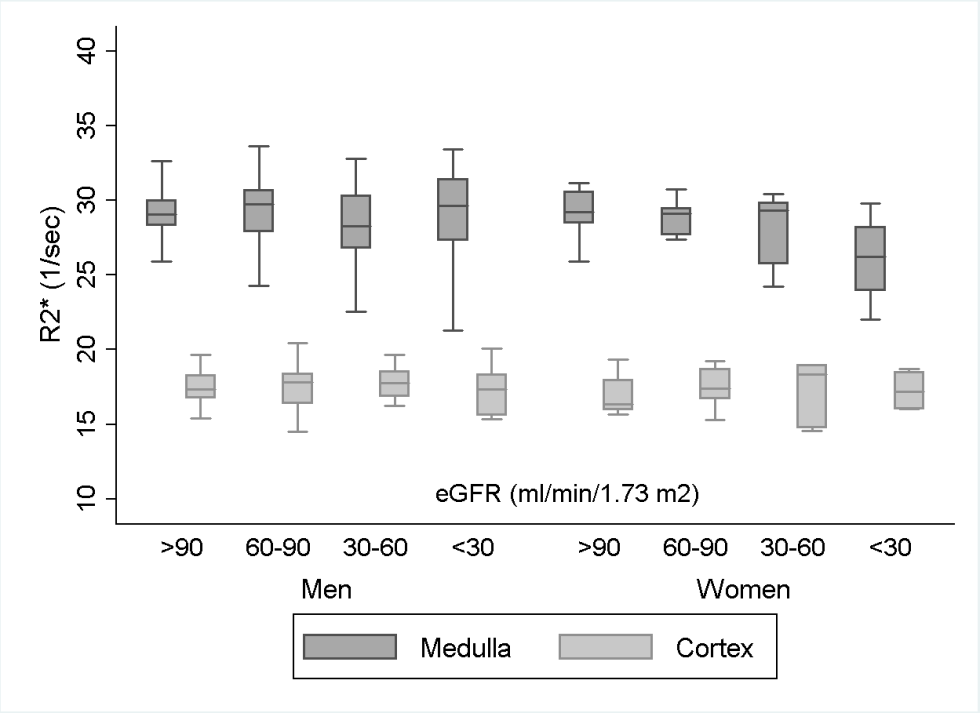

Supplement: Figure S2 — Medullary and cortical R2* values over decreasing eGFRmdrd values, by gender. The number of subjects was n = 44 (for the eGFR>90 ml/min/1.73 m2 category), 41 (eGFR 60–90), 25 (eGFR 30–60), 15 (eGFR <30) in men and respectively 30,24,11, and 5 in women. (TIF) [file pone.0095895.s002.tif]
