# Supplementary material for: Determinants of Renal Tissue Oxygenation as Measured with BOLD-MRI in Chronic Kidney Disease and Hypertension in Humans
Source: PLoS One. 2014 Apr 23;9(4):e95895. doi: 10.1371/journal.pone.0095895 (PMC3997480; doi:10.1371/journal.pone.0095895)
Supplement: Table S1 — Drug treatment of patients across different stages of chronic kidney disease (CKD). (DOCX) [file pone.0095895.s003.docx]

**Supplementary Table S1:** Drug treatment across different stages of CKD.

| **Stages of CKD** | **I** | **II** | **III** | **IV** | **V** |
| --- | --- | --- | --- | --- | --- |
| **n per CKD category (total=95)** | 20 | 20 | 35 | 15 | 5 |
| *Antihypertensive medication (%)* |  |  |  |  |  |
| Beta blocker | 21 | 26 | 43 | 43 | 50 |
| Alpha blocker | 5 | 0 | 3 | 0 | 0 |
| ACE-inhibitor | 21 | 11 | 17 | 21 | 25 |
| AT-II type 1 receptor blocker | 32 | 53 | 63 | 64 | 25 |
| Calcium channel blockers | 16 | 26 | 37 | 57 | 25 |
| Thiazide diuretic | 26 | 53 | 13 | 29 | 25 |
| Loop diuretic | 11 | 0 | 17 | 43 | 50 |
| *Cholesterol lowering medication* |  |  |  |  |  |
| Statine | 37 | 42 | 60 | 93 | 75 |
| Fibrate | 0 | 0 | 7 | 0 | 0 |
| *Antiplatelet agent* |  |  |  |  |  |
| Aspirin | 11 | 32 | 27 | 57 | 75 |
| *Uric acid lowering medication* |  |  |  |  |  |
| Allopurinol | 0 | 16 | 20 | 36 | 25 |
| *Vitamin D* | 25 | 26 | 27 | 29 | 50 |
|  | |  |  |  |  |
| All values are expressed as the percentage of patients of each group treated with the drug in question | |  |  |  |  |
