# Supplementary material for: Determinants of Renal Tissue Oxygenation as Measured with BOLD-MRI in Chronic Kidney Disease and Hypertension in Humans
Source: PLoS One. 2014 Apr 23;9(4):e95895. doi: 10.1371/journal.pone.0095895 (PMC3997480; doi:10.1371/journal.pone.0095895)
Supplement: Table S2 — Multivariate linear regression analysis examining associations between baseline characteristics and medullary R2* levels. Associations between medullary R2* levels and baseline characteristics are expressed as regression coefficient β (95% CI). (DOCX) [file pone.0095895.s004.docx]

**Supplementary Table S2**: Multivariate linear regression analysis examining correlations between baseline characteristics and medullary R2* levels, expressed as regression coefficient β (95% CI).

| **Medullary R2*** | **Age, sex adjusted** | | | | **Fully adjusted¹** | | | |
| --- | --- | --- | --- | --- | --- | --- | --- | --- |
|  |  | | | |  | | | |
|  | **β** | **95% CI** | | ***p*** | **β¹** | **95% CI** | | ***P*** |
|  |  |  | |  |  |  | |  |
| Sex (female vs. male) | -0.58 | -0.05 | 0.01 | 0.15 | -0.43 | -1.66 | 0.79 | 0.49 |
| Age (per year) | -0.02 | -0.06 | 0.01 | 0.08 | -0.01 | -0.05 | 0.03 | 0.72 |
| BMI (per kg/m2) | -0.03 | -0.10 | 0.05 | 0.50 | -0.03 | -0.13 | 0.08 | 0.61 |
| eGFR (MDRD) | 0.01 | -0.01 | 0.02 | 0.26 | -0.003 | -0.02 | 0.02 | 0.75 |
| Smoking (yes vs. no) | 0.29 | -0.65 | 1.24 | 0.54 | 0.28 | -0.95 | 1.40 | 0.77 |
| Urinary 24h sodium excretion (mmol) | 0.001 | -0.004 | 0.006 | 0.70 | 0.002 | -0.004 | 0.008 | 0.52 |
| Diabetes (yes vs. no) | -0.79 | -1.87 | 0.28 | 0.15 | -1.01 | -2.60 | 0.58 | 0.41 |

^1^ adjusted for gender, age, BMI, eGFR, smoking, urinary sodium excretion, Hb, and diabetes
