# Supplementary material for: Determinants of Renal Tissue Oxygenation as Measured with BOLD-MRI in Chronic Kidney Disease and Hypertension in Humans
Source: PLoS One. 2014 Apr 23;9(4):e95895. doi: 10.1371/journal.pone.0095895 (PMC3997480; doi:10.1371/journal.pone.0095895)
Supplement: Table S3 — Multivariate linear regression analysis examining associations between baseline characteristics and cortical R2* levels. Associations between cortical R2* levels and baseline characteristics are expressed as regression coefficient β (95% CI). (DOCX) [file pone.0095895.s005.docx]

**Supplementary Table S3**: Multivariate linear regression analysis examining correlations between baseline characteristics and cortical R2* levels, expressed as regression coefficient β (95% CI).

| **Cortical R2*** | **Age, sex adjusted** | | | | **Fully adjusted¹** | | | |
| --- | --- | --- | --- | --- | --- | --- | --- | --- |
|  | **β** | **95% CI** | | ***p*** | **β¹** | **95% CI** | | ***p*** |
| Sex (female vs. male) | -0.81 | -1.72 | 0.11 | 0.09 | -1.80 | -3.20 | -0-37 | *0.01* |
| Age (per year) | 0.02 | -0.01 | 0.05 | 0.29 | -0.01 | -0.06 | 0.04 | 0.70 |
| BMI (per kg/m2) | -0.04 | -0.13 | 0.06 | 0.43 | -0.04 | -0.16 | 0.09 | 0.58 |
| eGFR (MDRD) | -0.01 | -0.03 | 0.01 | 0.23 | -0.01 | -0.04 | 0.01 | 0.29 |
| Smoking (yes vs. no) | 0.87 | -0.22 | 1.97 | 0.12 | 1.04 | -0.42 | 2.50 | 0.16 |
| Urinary 24h sodium excretion (mmol) | -0.007 | -0.013 | -0.006 | 0.03 | 0.004 | -0.01 | 0.002 | 0.13 |
| Diabetes (yes vs. no) | 1.02 | -0.23 | 2.27 | 0.11 | 1.67 | -0.15 | 3.48 | 0.07 |
|  |  |  |  |  |  |  |  |  |

^1^ adjusted for gender, age, BMI, eGFR, smoking, urinary sodium excretion, Hb, and diabetes.
