# Supplementary material for: Determinants of Renal Tissue Oxygenation as Measured with BOLD-MRI in Chronic Kidney Disease and Hypertension in Humans
Source: PLoS One. 2014 Apr 23;9(4):e95895. doi: 10.1371/journal.pone.0095895 (PMC3997480; doi:10.1371/journal.pone.0095895)
Supplement: Table S4 — Multivariate linear regression analysis examining correlations between baseline characteristics and medullary and cortical R2* levels. This analysis includes the additional variables glycemia, serum uric acid level and 24 h urinary proteinuria. (DOCX) [file pone.0095895.s006.docx]

**Supplementary Table S4**: Multivariate linear regression analysis examining correlations between baseline characteristics and medullary and cortical R2* levels, expressed as regression coefficient β (95% CI). This analysis includes the additional variables glycemia, serum uric acid level and 24h urinary proteinuria.

|  | **Medullary R2*** | | | | **Cortical R2*** | | | |
| --- | --- | --- | --- | --- | --- | --- | --- | --- |
|  |  | | | |  | | | |
|  | **β¹** | **95% CI** | | ***p*** | **β¹** | **95% CI** | | ***p*** |
|  |  |  | |  |  |  | |  |
| Mean Arterial BP (per mmHg) | -0.04 | -0.82 | -0.003 | 0.036 | 0.003 | -0.005 | -0.06 | 0.91 |
| Urinary 24h protein excretion (per g) | -0.18 | -0.82 | 0.46 | 0.58 | -0.39 | -1.17 | 0.38 | 0.32 |
| RAAS-blocker (yes vs.no) | -0.86 | -1.90 | 0.18 | 0.11 | 0.34 | -0.92 | 1.60 | 0.60 |
| Serum glycemia (per mmol/l) | -0.11 | -0.44 | 0.23 | 0.54 | 0.91 | 0.54 | 1.28 | <0.001 |
| Serum uric acid (per µmol/l) | -0.0001 | -0.006 | 0.005 | 0.76 | 0.014 | 0.008 | 0.02 | <0.001 |

^1^ adjusted for gender, age, BMI, eGFR, smoking, urinary sodium excretion, Hb, and diabetes
