# Supplementary material for: Determinants of Renal Tissue Oxygenation as Measured with BOLD-MRI in Chronic Kidney Disease and Hypertension in Humans
Source: PLoS One. 2014 Apr 23;9(4):e95895. doi: 10.1371/journal.pone.0095895 (PMC3997480; doi:10.1371/journal.pone.0095895)
Supplement: Methods and Results S1 — Extended methods and results. - Screening. -Methods: Justification hydration protocol. -Results: * Furosemide-induced change in R2* in patients already on diuretics. * Sensitivity analysis in subgroup excluding healthy women <40 years and male CKD patients >75 years. (DOCX) [file pone.0095895.s007.docx]

**Extended methods supplement**:

**Screening:**

A total of 452 persons were screened; 172 refused, 53 were excluded (reasons: incapable of understanding the protocol, polycystic kidney disease, contra-indication for MRI), and 32 had insufficient quality of MR images, leaving 195 for final analysis.

**Methods:**

Since it has been shown previously that acute hydration conditions influence the BOLD-signal (Prasad, Epstein, Kidney International 1999, 55: p294-8), standardizing hydration status is important. The 'slow 5h hydration protocol' as used in this and previous studies was chosen for practical reasons. The aim was to standardize fluid intake in all participants, and study them as much as possible under 'real life' chronic conditions. For this reason, performing BOLD-MRI in non-hydrated or acutely hydrated (as with the Prasad protocol: 20ml/kg in 15 minutes) subjects, seemed less feasible or physiological. In order to assess whether this hydration protocol induced a similar 'wash out' effect (hence: a decrease in R2* of -3.2 sec^-1^  at 1.5T) as the acute hydration protocol previously described by Prasad, we performed a cross-over study in 9 healthy individuals (6 men and 3 women, aged 35±11y, BMI 24±2 kg/m^2^). All individuals underwent once a BOLD-MRI (at 3T) without prehydration (1.5ml/kg was drunk at 8am, nothing thereafter) and once after our 5h hydration protocol (5ml/kg at 8 am, followed by 3ml/kg every hour till 12 am). Time between the two MRI's was at most one week.

Individual medullary and cortical R2* values are shown in the table. There were no significant differences in R2* between the hydration or dehydration phase (mean(±SD) medulla R2* dehydrated vs hydrated: respectively 29.0±2.5 sec^-1^ vs 29.7±1.7 sec^-1^, p=0.2; cortex: 16.6±1.0 sec^-1^ vs 16.5±1.3 sec^-1^, p=0.91). Therefore, our hydration protocol allowed to avoid differences in prehydration between the individuals, without inducing large acute changes in R2*.

| **R2*** | **Medulla** |  | **Cortex** |  |
| --- | --- | --- | --- | --- |
|  | *dehydrated* | *hydrated* | *dehydrated* | *hydrated* |
| Female | 28.25 | 28.28 | 17.35 | 18.08 |
| Female | 24.65 | 27.94 | 15.17 | 15.92 |
| Female | 30.04 | 31.15 | 16.99 | 18.25 |
| Male | 33.2 | 32.8 | 17.7 | 16.76 |
| Male | 28.02 | 28.11 | 16.63 | 15.99 |
| Male | 26.36 | 28.57 | 15.24 | 14.12 |
| Male | 29.72 | 30.55 | 16.07 | 16.03 |
| Male | 30.86 | 29.54 | 17.85 | 17.44 |
| Male | 30.25 | 30.44 | 16.14 | 16.28 |
| **Mean** | **29.0±2.5** | **29.7±1.7** | **16.6±1.0** | **16.5±1.3** |

**Results**:

Furosemide-induced change in R2* in patients already on diuretics:

The effect of furosemide was smaller in the 15 CKD patients already on chronic loop diuretics: median decrease in R2* 1.8 sec^-1^ (0.49; 3.1), versus 3.6 sec^-1^ (2.7; 4.9) in the 71 CKD patients not on loop diuretics (p<0.01). This difference was partly explained by a difference in kidney function (median eGFR_mdrd_ 27.3 ml/min/1.73 m² in those on loop diuretics versus 59ml/min/1.73 m² in those who were not). Nevertheless, in multivariable linear regression analysis, adjusted for age and gender the R2*response to furosemide remained significantly correlated with eGFR_mdrd_ when loop diuretics were included in the model (adjusted β per ml/min/1.73m²: -0.028 (95% CI 0.01; 0.046), p=0.004). There were no correlations between the cortical response to furosemide and eGFR, nor were there any significant differences in the response to furosemide whether or not CKD patients were on thiazide-diuretics or RAAS-blockers.

In hypertension, only two patients were on chronic loop diuretics. These patients had a lower mean decrease in medullary R2* after furosemide than hypertensives not on loopdiuretics (2.57±0.03 vs 4.84±2.1 sec^-1^). A total of twelve patients were on thiazides; the effect of furosemide was smaller in hypertensive patients on thiazides, yet this difference did not reach statistical significance (3.84±1.9 vs 4.96±2.1 sec^-1^).

Sensitivity analysis:

Sensitivity analyses were performed in a subgroup of participants (n=177); in this analysis, healthy women aged <40 years (n=13) were excluded from the control group, and male patients aged >75 years from the CKD group (n=5). There were no significant differences in age and sex between the selected groups as shown in table 1.

| **Table 1 Total (n=177)** | **Control (n=29)** | **CKD (n=90)** | **AHT (n=58)** |
| --- | --- | --- | --- |
| Age (years) | 51±12 | 54±14 | 57±11 |
| Sex (% female) | 35 | 31 | 32 |
| Currently smoking (%) | 10* | 28 | 35 |
| Body Mass Index (kg/m2) | 26±5 | 28±4 | 29±5 |
| Systolic BP (mmHg) | 125±13* | 135±19 | 142±16** |
| Diastolic BP (mmHg) | 75±11 | 77±12 | 82±10** |
| eGFR (CKD-EPI, ml/min/1.73m^2^) | 93±14* | 58±31 | 91±15** |
| Hemoglobin (g/dl) | 139±9* | 132±18 | 138±13** |
| Blood glucose (mmol/l) | 5.8±0.8 | 6.4±2.0 | 6.1±1.2 |
| Diabetes (%) | 0* | 23 | 17 |
| Blood potassium (mmol/l) | 3.9±0.2* | 4.2±0.6 | 3.8±0.3** |
| Venous bicarbonate (mmol/l) | 27(24;30)* | 25 (14;32) | 27 (23;32)** |
| Blood uric acid (μmol/l) | 305 (130;450)* | 387 (168;662) | 342 (163;548)** |
| Oxygen saturation (%) | 96±2.0 | 96±1.9 | 96±1.6 |
| 24h Urinary volume (ml) | 1715 (694;5008) | 2068 (585;4356) | 1812 (780;3945) |
| 24h Urinary sodium excretion (mmol) | 164±74 | 172±94 | 174±87 |
| 24h Urinary protein excretion (g) | 0.06 (0;0.12)* | 0.3 (0;9.4) | 0.07 (0;0.17)** |
| 24h Urinary albumin excretion (mg) | 4.0 (0;23)* | 97 (1;6131) | 10 (0;29)** |
| 24h Urinary creatinine clearance (ml/min) | 126 (83;213)* | 68 (14; 170) | 115 (73;249)** |

* p<0.05, control vs CKD; **p<0.05, AHT vs CKD

Furosemide-induced changes in cortical and medullary R2* values are shown in the table 2 below.

| **Table 2 N=177** | **Control (n=29)** | **CKD (n=90)** | **AHT (n=58)** | **p (ANOVA)** |
| --- | --- | --- | --- | --- |
|  |  |  |  |  |
| **Baseline Medullary R2*** | **29.3±2.4** | **28.8±2.6** | **28.6±2.1** | 0.44 |
| men | 29.5±2.8 | 29.1±2.6 | 28.7±2.1 |  |
| women | 29.1±1.1 | 28.3±2.7 | 28.4±2.2 |  |
| **Furosemide-induced Change medullary R2*** | **-5.9±3.0** | **-3.8±2.0** | **-4.8±2.1** | <0.01 |
| men | -6.4±3.1 | -3.8±2.6 | -4.9±2.1 |  |
| women | -4.8±2.7 | -3.7±2.1 | -4.2±2.1 |  |
| **Baseline Cortical R2*** | **17.2 (16.3;18.8)** | **17.8 (16.4;18.5)** | **17.4 (16.3;18.3)** | 0.48 |
| men | 17.7 (16.9;19.0) | 17.8 (16.9;18.5) | 17.4 (16.4;18.2) |  |
| women | 16.3 (15.7;17.3) | 17.5 (16.1;18.7) | 17.4 (16.0;18.6) |  |

Furosemide-induced change in medullary R2* was still larger in men in the control group and AHT group, yet without reaching statistical significance. Multivariate analysis are shown in table 3.

| **Table 3** | **Medullary R2*** | | **Cortical R2*** | |
| --- | --- | --- | --- | --- |
|  |  | |  |  |
|  |  | |  |  |
|  | **β¹** | ***P*** | **β¹** | ***p*** |
|  |  |  |  |  |
| Sex (female vs. male) | -0.26 | 0.69 | -1.5 | 0.06 |
| Age (per year) | 0.001 | 0.96 | -0.02 | 0.48 |
| BMI (per kg/m2) | -0.01 | 0.98 | -0.05 | 0.94 |
| eGFR (MDRD) | 0.007 | 0.49 | -0.01 | 0.27 |
| Smoking (yes vs. no) | 0.04 | 0.77 | 0.91 | 0.25 |
| Urinary 24h sodium excretion (mmol) | 0.001 | 0.74 | 0.006 | 0.1 |
| Diabetes (yes vs. no) | -1.05 | 0.19 | 1.67 | 0.09 |

^1^ adjusted for gender, age, BMI, eGFR, smoking, urinary sodium excretion, Hemoglobin, and diabetes

The main conclusions of the subgroup-analysis were similar as those found in the whole study population: no difference in cortical or medullary R2* levels between the groups, a blunted R2* response to furosemide in CKD and hypertensive patients as compared with healthy controls, no association between markers of kidney function and renal R2* levels in multivariate linear regression analysis, and a positive association between cortical R2* levels, glycemia and uric acid. There was still a trend towards higher cortical R2* levels in male participants, suggesting lower oxygenation in men (see table 2), yet the association between gender and cortical R2* was no longer significant in multivariate regression analysis (see table 3, β= -1.5, p=0.06), possibly due to the low number of remaining women.
